# Supplementary material for: Complexome profiling of the Chlamydomonas psb28 mutant reveals TEF5 as an early PSII assembly factor
Source: Plant Cell. 2025 Mar 17;37(6):koaf055. doi: 10.1093/plcell/koaf055 (PMC12164590; doi:10.1093/plcell/koaf055)
Supplement: koaf055_Supplementary_Data [file koaf055_supplementary_data.zip › psb28_interactive.zip!/plots/intensity/qualityPlots/peptides/NUOB13_Cre13.g568800.t1.2.html]

### Cre13.g568800.t1.2

Trivial name: NUOB13  
SeqCoverage [median]: 30.30  
Mass: 17.95 kDa  
  
 

| Sequence | MissedCleavage | Mass [Da] | wtIntensity | muIntensity | Score |
| --- | --- | --- | --- | --- | --- |
| AAPEGSDYR | 0 | 964.43 | 1279.58 | 2899.37 | 102.51 |
| AAPEGSDYRK | 1 | 1092.52 | 3833.94 | 4569.90 | 82.75 |
| AVADVEIDFNNR | 0 | 1361.66 | 1637.08 | 411.89 | 126.31 |
| DHLSKEPGTR | 1 | 1138.57 | 4941.28 | 2572.00 | 61.21 |
| LIEALTEVLSSIK | 0 | 1414.83 | 5721.24 | 0.00 | 157.68 |
| YVGIRDHLSKEPGTR | 2 | 1726.91 | 2317.67 | 1918.37 | 47.77 |
|  |  |  | 19730.8 | 12371.5 |  |
